# Outpatient total knee and hip arthroplasty present comparable and even better clinical outcomes than inpatient operation

**DOI:** 10.3389/fsurg.2022.833275

**Published:** 2022-09-06

**Authors:** Song Gong, Yihu Yi, Ruoyu Wang, Lizhi Han, Tianlun Gong, Yuxiang Wang, Wenkai Shao, Yong Feng, Weihua Xu

**Affiliations:** ^1^ Department of Orthopedics, The Central Hospital of Wuhan, Tongji Medical College, Huazhong University of Science and Technology, Wuhan 430014, China; ^2^ Department of Orthopedics, Union Hospital, Tongji Medical College, Huazhong University of Science and Technology, Wuhan 430022, China

**Keywords:** outpatient total knee arthroplasty, outpatient total hip arthroplasty, complications, readmissions, reoperations

## Abstract

**Background:**

The purpose of this study was to compare total complications, complications stratified by type, readmissions, and reoperations at 30 and 90 days after outpatient and standard inpatient total knee and total hip arthroplasty (TKA, THA).

**Methods:**

A literature search was conducted from the PubMed, Cochrane Library, and Embase databases for articles published before 20 August 2021. The types of studies included prospective randomized controlled trials, prospective cohort studies, retrospective comparative studies, retrospective reviews of THA and TKA registration databases, and observational case-control studies. Comparisons of interest included total complications, complications stratified by type, readmissions, and reoperations at 30 and 90 days. The statistical analysis was performed using Review Manager 5.3.

**Results:**

Twenty studies with 582,790 cases compared relevant postoperative indicators of outpatient and inpatient total joint arthroplasty (TJA) (TKA and THA). There was a significant difference in the total complications at 30 days between outpatient and inpatient THA (*p* = 0.001), readmissions following TJA (*p* = 0.03), readmissions following THA (*p* = 0.001), stroke/cerebrovascular incidents following TJA (*p* = 0.01), cardiac arrest following TJA (*p* = 0.007), and blood transfusions following TJA (*p* = 0.003). The outcomes showed an obvious difference in 90-day total complications between outpatient and inpatient TJA (*p* = 0.01), readmissions following THA (*p* = 0.002), and surgical-related pain following TJA (*p *< 0.001). We did not find significant differences in the remaining parameters.

**Conclusion:**

Outpatient procedures showed comparable and even better outcomes in total complications, complications stratified by type, readmissions, and reoperations at 30 and 90 days compared with inpatient TJA for selected patients.

## Introduction

The number of total knee arthroplasty (TKA) and total hip arthroplasty (THA) procedures has increased significantly over the last two decades (1–3). The number of total joint arthroplasty (TJA) (TKA and THA) procedures is expected to reach 4 million by 2,030 in the United States (4). Advances in surgical techniques, perioperative anesthesia, multimodal pain management, and accelerated rehabilitation have led to substantial reductions in the average hospital length of stay (LOS) (5–8). TJA is increasingly being performed in outpatient settings, including hospital outpatient departments (HOPDs) and ambulatory surgery centers (ASCs), to shorten the hospital LOS, reduce the pressure from payers, control the overall cost, and allow patients to return to activities early (9–12). Although outpatient TJA is becoming more common, the frequency with which it is performed remains very low due to concerns about the safety of outpatient surgery (13–15). The acceptable outpatient TJA safety is to ensure that the rate of postoperative complications is basically the same as that in inpatients. To reduce the considerable amount of medical expenses associated with TJA, it is essential that the hospital LOS be shortened and the rate of complications be controlled. Therefore, controlling and reducing the rate of postoperative complications has been the focal point of outpatient TJA (13, 15–17). Published studies have presented conflicting results regarding postoperative complications. Several studies (13, 18, 19) have reported that outpatient TJA is associated with a high rate of perioperative complications. Some researchers have concluded that outpatient TJA is safe and feasible for selected healthy patients, with outcomes comparable to those of standard inpatient surgery (10, 20–22). Some studies have even shown that compared with inpatient TJA, outpatient TJA reduces the rate of complications and readmissions (20, 23–25). In addition, the cost savings of outpatient TJA are noteworthy (21, 26, 27). Several studies have shown that outpatient TJA can save between $4,000 and $8,000 per case (26, 28). Several studies have reported that patients have higher satisfaction with outpatient operations than with inpatient operations (24, 29, 30). We expect to conclude that outpatient procedures will have comparable total complications, complications stratified by type, readmissions, and reoperations at 30 and 90 days compared with inpatient TJA. This is the first study including the most recent literature and large-volume cases to present comprehensive information on the total complications, complications stratified by type, readmissions, and reoperations.

In this study, a meta-analysis was conducted to compare the total complications, complications stratified by type, readmissions, and reoperations at 30 and 90 days after outpatient and standard inpatient TJA. The types of studies included prospective randomized controlled trials, prospective cohort studies, retrospective comparative studies, retrospective reviews of THA and TKA registration databases, and observational case-control studies. We presumed the security of outpatient TJA to be comparable to that of inpatient surgery for selected patients.

## Materials and methods

### Search strategy

A literature search was conducted with the PubMed, Cochrane Library, and Embase databases. This work has been reported in line with the Preferred Reporting Items for Systematic Reviews and Meta-Analyses (PRISMA) and Assessing the Methodological Quality of Systematic Reviews (AMSTAR) guidelines (31). Our work has been registered in the PROSPERO international prospective register of systematic reviews (registration number CRD42020180124). The literature search was restricted to articles published in the English language before 20 August 2021. The Cochrane Central Register of Controlled Studies was searched using the following terms: outpatient, ambulatory surgery, day surgery, inpatient, total joint arthroplasty (TJA) or total joint replacement (TJR), total knee arthroplasty (TKA) or total knee replacement (TKR), and total hip arthroplasty (THA) or total hip replacement (THR).

### Inclusion and exclusion criteria

The eligibility criteria for this study were as follows:

1. Studies that included patients undergoing TKA because of a disease such as osteoarthritis, rheumatoid arthritis, or posttraumatic arthritis. Studies that included patients undergoing THA because of a disease such as femoral head necrosis, femoral neck fracture, osteoarthritis, rheumatoid arthritis, posttraumatic arthritis, or congenital hip dysplasia.
2. Prospective randomized controlled trials, prospective cohort studies, retrospective comparative studies, retrospective reviews of THA and TKA registration databases, and observational case-control studies.
3. Studies comparing outpatient procedures with inpatient TKA or THA.
4. Studies that included cohorts matched and adjusted for age, comorbidities and anesthesia grade of outpatients and inpatients without significant differences.
5. Studies that included postoperative evaluation indicators, including at least one of the following: total complications, complications stratified by type, readmissions, or reoperations.
6. A representative article was selected if several studies referred to the same database, and the remaining studies were excluded for reasons of avoiding repetition.

### Data extraction

Two independent reviewers extracted the data according to the abovementioned inclusion and exclusion criteria. Disagreements between reviewers were resolved by consultation with senior reviewers. The demographics and characteristics of the studies included first author, age, year of publication, study period, country, study type or source, follow-up time, outpatient definition, type of surgery, number of total patients, number of outpatients, and number of inpatients. The comparisons of interest included total complications, complications stratified by type, readmissions, and reoperations at 30 and 90 days. The complications stratified by type included surgical site infection, pneumonia, renal insufficiency, renal failure, urinary tract infection, stroke/cerebrovascular incidents, cardiac arrest, myocardial infarction, blood transfusion, sepsis/septic shock, deep vein thrombosis, revision, periprosthetic fracture, surgical-related pain and arthrofibrosis.

### Statistical analysis

The odds ratio (OR) was used to assess the effect, and the Mantel–Haenszel (MH) statistical method was selected because all data were dichotomous variables, and this study involved randomized controlled trials, prospective studies, retrospective studies, etc. A fixed-effects model was used when there was low heterogeneity among studies (*p *> 0.10 and *I*^2^ < 25%); otherwise, a random-effects model was used. Publication bias was evaluated by funnel plots. Sensitivity analysis was conducted by a leave-one-out analysis. The statistical analysis was performed using Review Manager 5.3 (Copenhagen: The Nordic Cochrane Centre, The Cochrane Collaboration, 2014), and *p *< 0.05 indicated a significant difference.

## Results

**Figure 1:**
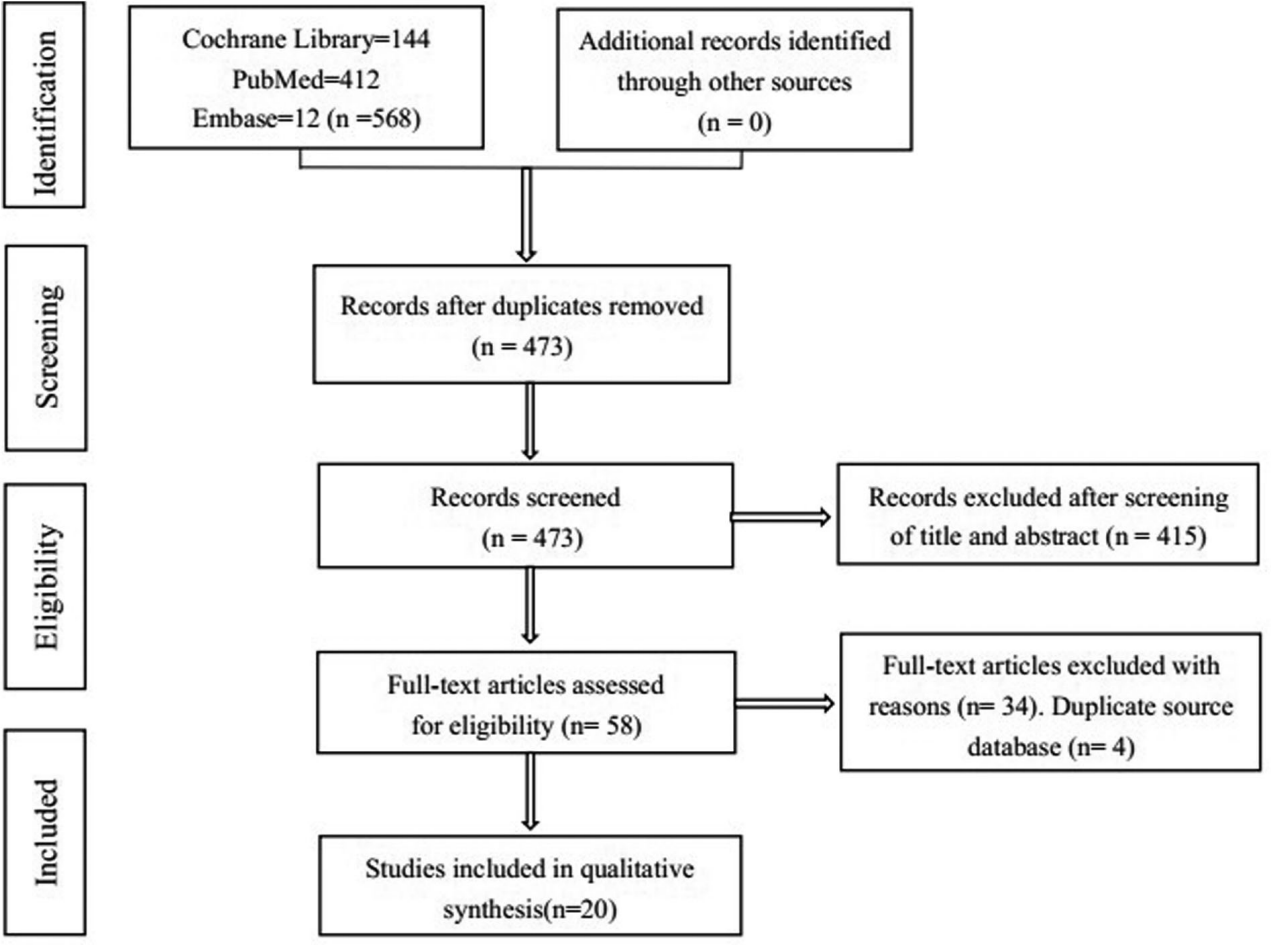
Flow charts of the study selection process for the meta-analysis of outpatient vs inpatient TJA.

There were 568 articles retrieved by searching the PubMed, Cochrane Library, and Embase databases, and no additional articles were found through manual searching. We removed 95 duplicate records using literature management software. A total of 357 records were excluded after the titles and abstracts were strictly screened. Finally, 20 articles (18, 19, 21, 26, 32–47) were included in our meta-analysis after the full texts were read and duplicate studies using the same source dataset were excluded. A flow chart of the study selection process is illustrated in Figure 1. A total of 582,790 patients who underwent TKA or THA were included in this study. The demographics and characteristics of the studies involved in the systematic review and meta-analysis are presented in Table 1.

**Table 1:** Demographics and characteristics of the studies included in the systematic review and meta-analysis.

First author		Year	Study period	Country	Study Type or Source		Follow up	Outpatient definition
Arshi (24)	2017		2007–2015	USA	RS, HPPRD	30		Discharge within 24 h
Arshi (3)	2019		2007–2016	USA	RS, HPIRD	30		Discharge within 24 h
Aynardi (27)	2014		2008–2011	USA	OCCS	90		Discharge within 23 h
Bovonratwet (21)	2017		2005–2014	USA	ACS-NSQIP	30		LOS = 0 days
Carey (13)	2020		2014–2016	USA	THAMCCED	30,90		NS
Cassard (6)	2018		2014.04–2017.07	France	RCS	30		NS
Coenders (12)	2020		2014.04–2017.10	Netherlands	PCS	90		Same-day discharge
Courtney (31)	2018		2014.01–2015.12	USA	RS, ACS-NSQIP	30		LOS = 0 days
Darrith (45)	2019		2013.01–2016.06	USA	RS	90		Same-day discharge
Gauthier-Kwan (14)	2018		2010.09–2015.05	Canada	PCCS	90		Same-day discharge
Gogineni (15)	2019		2016.12–2018.03	USA	RS	90		Same-day discharge
Goyal (46)	2017		2014.07–2015.09	USA	PRCT	28		Discharge within 12 h
Greenky (19)	2019		2015–2016	USA	ACS-NSQIP	30		Same-day discharge
Gromov (7)	2019		2015.12–2017.09	Denmark	PCS	90		Same-day discharge
Kolisek (22)	2009		2004.01–2006.07	USA	PS	90		Discharge within 23 h
Lovald (40)	2014		1997–2009	USA	RS, LDS	90		NS
Nelson (25)	2017		2005–2014	USA	RS, ACS-NSQIP	30		LOS = 0 days
Richards (8)	2018		2014.03–2017.08	Canada	RS	90		Same-day discharge
Springer (4)	2017		2010.09–2011.05	USA	RS	30		Same-day discharge
Weiser (47)	2018		2014.01–2016.12	USA	RS	30,90		Same-day discharge

First author	Age		Total Patients	Number of patients		Type of Surgery	TKA and/or THA(No.)	
	Outpatient	Inpatient		Outpatient	Inpatient		Outpatient	Inpatient
Arshi	NS	NS	133,342	4391	128,951	TKA	4391	128,951
Arshi	65–69^a^	70–74^a^	75,780	2184	73,596	THA	2184	73,596
Aynardi	59 ± 5.8	61. 5 ± 13.2	197	119	78	THA	119	78
Bovonratwet	64	67	112,922	642	112,280	TKA	642	112,280
Carey	55–64	55–64	5924	1481	4443	TKA, THA	858TKA & 623THA	2574TKA & 1869THA
Cassard	65.4 (44–78)	70.5 (47–86)	574	61	513	TKA	61	513
Coenders	63.7(58.8–67.7)	NS	607	217	390	THA	217	390
Courtney	72.3 ± 5.9	NS	49,136	365	48,771	TKA	365	48,771
Darrith	NS	NS	238	119	119	TKA, THA	46TKA & 73THA	46TKA & 73THA
Gauthier-Kwan	62.5 (50.4–75.0)	62.5 (51.2–74.0)	86	43	43	TKA	43	43
Gogineni	57.3 (24–80)	53.9	241	105	136	TKA, THA	56 TKA & 49THA	136 THA & TKA
Goyal	59.8 ± 8.5	60.2 ± 8.9	220	112	108	THA	112	108
Greenky	71.4 ± 5.2	NS	34,416	310	34,106	THA	310	34,106
Gromov	61 ± 11	62 ± 10.4	455	116	339	TKA, THA	46 TKA & 70THA	134 TKA & 205THA
Kolisek	55(42–64)	55(42–63)	128	64	64	TKA	64	64
Lovald	NS	NS	102,684	454	102,230	TKA	454	102,230
Nelson	NS	NS	63,844	420	63,424	THA	420	63,424
Richards	53.15 ± 10.18	50.98 ± 10.18	274	137	137	THA	137	137
Springer	61 (28–84)	65 (39–87)	243	137	106	TKA, THA	92TKA & 45THA	74TKA & 32THA
Weiser	56.8 ± 8.0	58.0 ± 11.2	1479	164	1315	THA	164	1315

RS, Retrospective study; HPPRD, The Humana subset of the PearlDiver Patient Record Database; HPIRD, The Humana subset of the PearlDiver Insurance Records Database; OCCS, Observational, case-control study; ACS-NSQIP, The American College of Surgeons National Surgical Quality Improvement Program; THAMCCED, The Truven Health Analytics MarketScan Commercial Claims and Encounters database; RCS, Retrospective comparative study; PCS, Prospective cohort study; PCCS, Prospective comparative cohort study; PRCT, Prospective randomized controlled trial; PS, Prospective study; LDS, The Medicare 5% Limited Data Set; h, Hours; LOS, Length of stay; NS, Not specified. TKA, Total knee arthroplasty; THA, Total hip arthroplasty; No, Number; MA, ^a^Median age; regarding the representation of age, a separate number represents the average age, A ± B represents the mean ± standard deviation, and A–B represents the age range; NS, Not specified.

### Comparison of 30-day total complications

**Figure 2:**
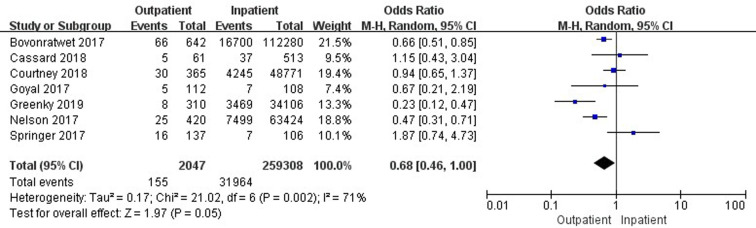
Comparison of 30-day total complications between outpatient and inpatient TJA.

**Figure 3:**
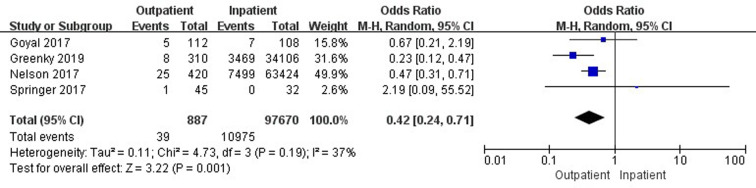
Comparison of 30-day total complications between outpatient and inpatient THA.

Seven studies (19, 33, 35, 37, 41, 42, 45) involving 261,355 cases compared 30-day total complications between outpatient and inpatient TJA. The results showed no significant difference in 30-day total complications between outpatient and inpatient TJA (95% confidence interval (CI) 0.46–1.00, *p* = 0.05) (Figure 2). Four studies (19, 33, 35, 37) involving 162,798 cases compared 30-day total complications between outpatient and inpatient TKA. There were no significant differences in 30-day total complications between outpatient and inpatient TKA (95% CI, 0.62–1.31, *p *= 0.58) (Supplementary Figure S1). Four studies (19, 41, 42, 45) including 98,557 cases compared 30-day total complications between outpatient and inpatient THA. Outpatient THA showed a significant advantage, as it was associated with fewer total complications than inpatient THA (95% CI, 0.24–0.71, *p *= 0.001) (Figure 3).

### Comparison of 30-day readmissions

**Figure 4:**
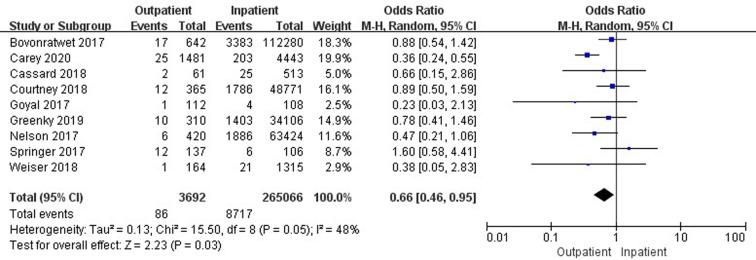
Comparison of 30-day readmissions between outpatient and inpatient TJA.

**Figure 5:**
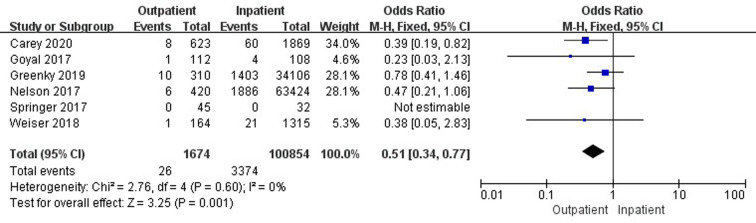
Comparison of 30-day readmissions between outpatient and inpatient THA.

Nine studies (19, 33–35, 37, 41, 42, 45, 47) involving 268,758 cases compared 30-day readmissions between outpatient and inpatient TJA. Outpatient TJA presented an obvious advantage, as it was associated with fewer readmissions than inpatient TJA (95% CI, 0.46–0.95, *p *= 0.03) (Figure 4). Five studies (19, 33–35, 37) involving 166,230 cases compared 30-day readmissions between outpatient and inpatient TKA. There was no significant difference in 30-day readmissions between outpatient and inpatient TKA (95% CI, 0.43–1.28, *p *= 0.29) (Supplementary Figure S2). Six studies (19, 34, 41, 42, 45, 47) including 102,528 cases compared 30-day readmissions between outpatient and inpatient THA. Outpatient THA showed an obvious advantage, as it was associated with fewer readmissions than inpatient THA (95% CI, 0.34–0.77, *p *= 0.001) (Figure 5).

### Comparison of 90-day total complications

**Figure 6:**
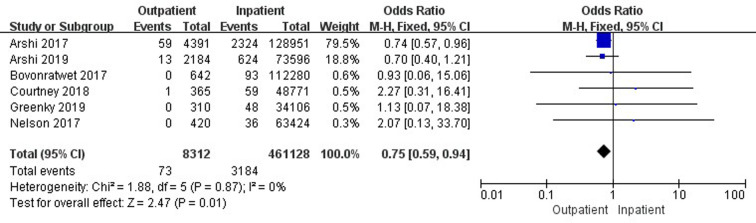
Comparison of 30-day stroke/cerebrovascular accidents between outpatient and inpatient TJA.

Nine studies (21, 26, 34, 36, 38–40, 44, 46) involving 110,379 cases reported a comparison of 90-day total complications between outpatient and inpatient TJA. There was a significant difference in 90-day total complications between outpatient and inpatient TJA (95% CI, 0.50–0.92, *p* = 0.01) (Figure 6). Five studies (21, 34, 38, 39, 44) involving 106,422 cases presented a comparison of 90-day total complications between outpatient and inpatient TKA. There was no significant difference in 90-day total complications between outpatient and inpatient TKA (95% CI, 0.52–1.36, *p *= 0.48) (Supplementary Figure S3). Five studies (26, 34, 36, 38, 46) including 3,716 cases compared 90-day total complications between outpatient and inpatient THA. There was no significant difference in 90-day total complications between outpatient and inpatient THA (95% CI, 0.31–1.12, *p *= 0.11) (Supplementary Figure S4).

### Comparison of 90-day readmissions

**Figure 7:**
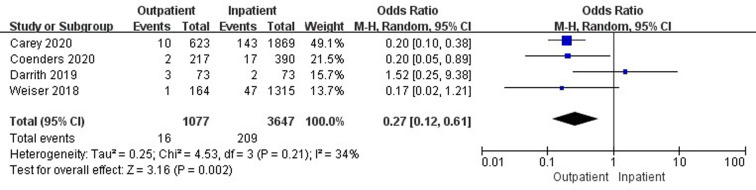
Comparison of 90-day readmissions between outpatient and inpatient THA.

Eight studies (26, 34, 36, 38–40, 43, 47) involving 111,714 cases reported a comparison of 90-day readmissions between outpatient and inpatient TJA. There was no significant difference in 90-day readmissions between outpatient and inpatient TJA (95% CI, 0.25–1.30, *p *= 0.18) (Supplementary Figure S5). Four studies (26, 34, 38, 39) involving 106,294 cases presented a comparison of 90-day readmissions between outpatient and inpatient TKA. There was no significant difference in 90-day readmissions between outpatient and inpatient TKA (95% CI, 0.17–2.66, *p *= 0.57) (Supplementary Figure S6). Four studies (34, 36, 38, 47) including 4,724 cases compared 90-day readmissions between outpatient and inpatient THA. Outpatient THA showed an obvious advantage, as it was associated with fewer readmissions than inpatient THA (95% CI, 0.12–0.61, *p *= 0.002) (Figure 7).

### Comparison of reoperations and complications stratified by type

**Figure 8:**
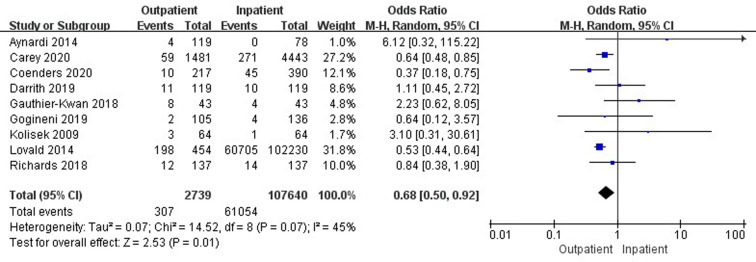
Comparison of 90-day total complications between outpatient and inpatient TJA.

**Figure 9:**
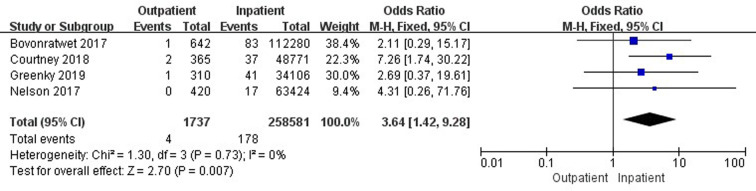
Comparison of 30-day cardiac arrests between outpatient and inpatient TJA.

**Figure 10:**
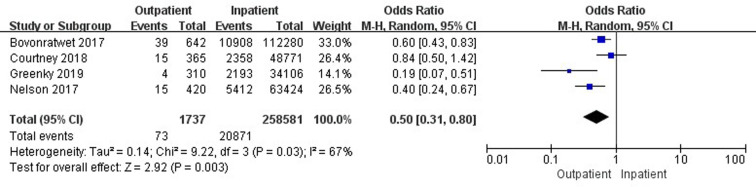
Comparison of 30-day blood transfusions between outpatient and inpatient TJA.

Six studies (18, 32, 33, 37, 42, 45) involving 469,440 cases compared 30-day stroke/cerebrovascular incidents between outpatient and inpatient TJA. There was a significant difference in 30-day stroke/cerebrovascular incidents between outpatient and inpatient TJA (95% CI, 0.59–0.94, *p *= 0.01) (Figure 8). Four studies (33, 37, 42, 45) involving 260,318 cases reported a comparison of 30-day cardiac arrest between outpatient and inpatient TJA. Inpatient TJA showed an obvious advantage, as it was associated with fewer cardiac arrests than outpatient TJA (95% CI, 1.42–9.28, *p *= 0.007) (Figure 9). Four studies (33, 37, 42, 45) involving 260,318 cases reported a comparison of 30-day blood transfusions between outpatient and inpatient TJA. Outpatient TJA showed an obvious advantage, as it was associated with fewer blood transfusions than inpatient TJA (95% CI, 0.31–0.80, *p *= 0.003) (Figure 10). Three studies (37, 41, 42) involving 83,772 cases reported a comparison of 30-day reoperations between outpatient and inpatient TJA. There was no significant difference in 30-day reoperations between outpatient and inpatient TJA (95% CI, 0.70–2.04, *p *= 0.51) (Supplementary Figure S7).

**Figure 11:**
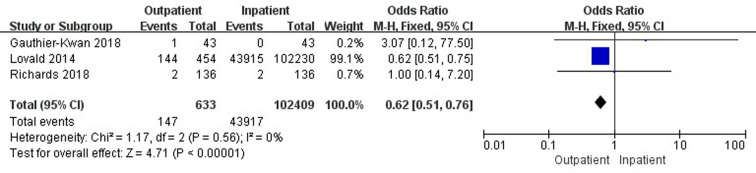
Comparison of 90-day surgical-related pain between outpatient and inpatient TJA.

Three studies (26, 39, 46) involving 103,042 cases reported a comparison of 90-day surgical-related pain between outpatient and inpatient TJA. Outpatient TJA showed an obvious advantage, as it was associated with fewer cases of surgical-related pain than inpatient TJA (95% CI, 0.51–0.76, *p *< 0.001) (Figure 11). Two studies (36, 38) involving 845 cases reported a comparison of 90-day reoperations between outpatient and inpatient TJA. There was no significant difference in 90-day reoperations between outpatient and inpatient TJA (95% CI, 0.07–8.56, *p *= 0.82) (Supplementary Figure S8).

There were no significant differences in 30-day cases of surgical site infection (95% CI, 0.35–1.16, *p *= 0.14) (Supplementary Figure S9), pneumonia (95% CI, 0.15–1.50, *p *= 0.21) (Supplementary Figure S10), renal insufficiency (95% CI, 0.52–5.12, *p *= 0.39) (Supplementary Figure S11), renal failure (95% CI, 0.68–8.36, *p *= 0.17) (Supplementary Figure S12), urinary tract infection (95% CI, 0.51–1.42, *p *= 0.55) (Supplementary Figure S13), myocardial infarction (95% CI, 0.72–1.80, *p *= 0.59) (Supplementary Figure S14), sepsis/septic shock (95% CI, 0.09–1.51, *p *= 0.17) (Supplementary Figure S15), or deep vein thrombosis (95% CI, 0.29–1.85, *p *= 0.51) (Supplementary Figure S16) between outpatient and inpatient TJA. There were no significant differences in cases of 90-day surgical site infection (95% CI, 0.44–1.28, *p *= 0.29) (Supplementary Figure S17), revision (95% CI, 0.42–2.08, *p *= 0.87) (Supplementary Figure S18), periprosthetic fracture (95% CI, 0.14–3.37, *p *= 0.64) (Supplementary Figure S19), deep vein thrombosis (95% CI, 0.23–1.62, *p *= 0.32) (Supplementary Figure S20), or arthrofibrosis (95% CI, 0.60–1.31, *p *= 0.55) (Supplementary Figure S21) between outpatient and inpatient TJA.

## Discussion

This study comprehensively analyzed total complications, complications stratified by type, readmissions, and reoperations at 30 and 90 days after outpatient and conventional inpatient procedures. The main finding was that outpatient procedures showed better results in THA total complications, THA readmissions, TJA readmissions, TJA stroke/cerebrovascular incidents, and TJA blood transfusion at 30 days postoperatively. Outpatient procedures presented fewer adverse events in regard to TJA total complications, THA readmissions, and TJA surgical-related pain at 90 days postoperatively compared with inpatient procedures.

There are several limitations in this study. First, the study presented significant potential bias. Outpatient protocols differed from inpatient protocols, and even outpatient protocols were not uniform. Some outpatient protocols followed enhanced recovery after surgery (ERAS) principles, while others were similar to inpatient protocols and tried to achieve same day discharge with strict patient selection. The selection criteria for outpatients and inpatients were inconsistent due to the different types of included studies. Second, according to the Improved Jadad Rating Scale score, only five prospective studies were included, and the remaining studies were retrospective or database studies. Relatively low-quality literature has limited persuasiveness. In the future, more multicenters, large-sample, randomized controlled trials will be needed to clarify the topic. Third, the definition of outpatient discharge time was inconsistent; it included same-day discharge, an LOS of 0 days, discharge within 12 h, discharge within 23 h, and discharge within 24 h. If the discharge time can be standardized, it will be of great benefit to the research on this topic.

Outpatient procedures have become a feasible treatment option and are gradually being performed more often based on substantial reductions in hospital LOS due to advances in surgical techniques, perioperative anesthesia, multimodal pain management, and accelerated rehabilitation (9–12). Common sense dictates that a prolonged LOS should provide a wider margin of security and lower the risk of complications. However, our results showed that the LOS of outpatient procedures was shortened and postoperative complications were reduced. This seemed to be slightly paradoxical. After a comprehensive analysis, we speculate that the possible reasons are as follows. First, the introduction of ERAS principles and innovation of technology and implants may lead to a shorter LOS and fewer complications in outpatient procedures (48–50). ERAS protocols require the collaboration of a multidisciplinary team, including surgeons, anesthesiologists, nurses, and physiotherapists, who follow specifically designed protocols on perioperative care and adjust their practices based on evolving scientific knowledge. Hence, it is possible for outpatients to have a shorter LOS and fewer complications than inpatients through multidisciplinary collaboration and delicacy management. Second, outpatient procedures may adhere to tighter patient selection criteria than inpatient procedures. Age is a crucial factor for outpatient and inpatient patient selection. Looking at the age comparison of the included literature, outpatient surgeons tend to choose younger patients. Another important factor is preoperative comorbidities; for example, high blood pressure, diabetes, and coronary heart disease. Outpatient surgeons tend to choose patients with fewer comorbidities. It is reasonable to consider that younger patients with fewer preoperative comorbidities could have a shorter LOS and fewer postoperative complications. Third, different anesthesia methods may affect early postoperative mobilization in outpatients and inpatients. Inpatients are more likely to receive general inhalation anesthesia, which is more likely to cause postoperative nausea, dizziness and vomiting and affect patients' early mobilization. However, outpatients generally receive spinal anesthesia, which can enable patients to mobilize early. Fourth, good preoperative education and home care are essential for the implementation of outpatient procedures. It is obvious that outpatient protocols address these two aspects better than inpatient programs.

The main obstacle to the implementation of outpatient TKA and THA came from the concerns of patients and surgeons regarding safety. The published literature showed opposite results regarding postoperative complications in outpatient and inpatient cohorts. Arshi et al. (24) showed that outpatient TKA was associated with a higher risk of postoperative 30-day complications, including surgical site infection, component failure, deep vein thrombosis, and knee stiffness, through a review of the Humana subset of the PearlDiver patient record database. However, several published studies showed that when performed in appropriately selected patients, outpatient TKA was not associated with a higher postoperative 30-day complication rate than inpatient TKA (6, 21, 31). Gogineni et al. (15) reported that outpatient TKA and THA in well-selected patients were feasible in an academic multidisciplinary tertiary care hospital, with postoperative 90-day complication rates approximating those of inpatient surgery. In addition, patients undergoing outpatient THA had no greater risk of postoperative 30-day complications than those who underwent inpatient surgery (3, 25). Some studies demonstrated that appropriately selected patients can undergo THA in an outpatient setting with no increase in complications at 90 days (8, 12, 27). Moreover, Greenky et al. (19) reported that outpatients and short-stay patients had lower 30-day complication rates than inpatients. Carey et al. (13) reported that outpatient procedures had a lower postoperative 90-day complication rate than inpatient TKA and THA. From a series of published studies, only one article reported that outpatient procedures have higher postoperative complications than inpatient surgeries, and two studies reported that the postoperative complications of outpatients are lower than those of inpatients. Most of the literature reported that outpatients and inpatients had comparable postoperative complications. After a comprehensive analysis, our results demonstrated that outpatient THA had fewer 30-day complications than the inpatient procedure, and outpatient TJA had fewer 90-day complications than inpatient surgery.

Readmission due to complications is the most direct cause of an increase in medical burden (34, 37, 42). Outpatient and inpatient TKA showed readmission rates of 1.98%–13.04% and 3.01%–8.11%, respectively, at 30 days (19, 33, 34). Outpatient and inpatient THA showed readmission rates of 0%–3.23% and 0%–4.11%, respectively, at 30 days (19, 42). Outpatient and inpatient TKA presented readmission rates of 0%–3.15% and 0%–9.87%, respectively, at 90 days (19, 34, 38). Outpatient and inpatient THA revealed readmission rates of 0.61%–4.11% and 2.74%–7.65%, respectively, at 90 days (34, 38, 47). We found an interesting phenomenon in which the maximum readmission rate at 30 days was higher than that at 90 days after TKA (13.04% vs. 9.87%). We performed a careful analysis and trusted the results. First, an inconsistency in the included articles was detected in the 30-day and 90-day groups because some articles reported the 30-day readmission data, and the other articles reported the 90-day outcomes. Second, the evidence may not be strong because of the small sample size of fewer than 100 cases (37). Therefore, a comprehensive analysis needs to be conducted in multiple studies with large sample sizes. Our study reported that outpatient TJA had fewer THA readmissions at 30 days and fewer THA readmissions at 90 days. Moreover, outpatient and inpatient TJA procedures showed comparable outcomes in TKA readmissions at 30 days and in TJA and TKA readmissions at 90 days. In summary, we conclude that outpatient TJA showed comparable and even better outcomes in readmissions at 30 and 90 days than did inpatient TJA.

Reoperations due to complications constitute the other direct cause of an increase in medical burden (37, 41, 42). Complications such as surgical site infection, periprosthetic fracture, and prosthesis dislocation are likely to require reoperation. In this study, the number of studies involving reoperations was a serious limitation, and it concluded that there was no significant difference within 30 and 90 days between the two groups. More evidence is needed to determine whether the two groups differ. Our analysis showed that outpatient TJA was associated with fewer stroke/cerebrovascular incidents than inpatient TJA. This result is possibly related to the lower average age and fewer comorbidities of outpatients (20). In this study, cardiac arrest was the only index in which outpatient TJA showed worse outcomes than inpatient TJA. This result is probably related to the lack of emergency medical support for outpatients at discharge. Our study showed that outpatient TJA required fewer blood transfusions than inpatient TJA. Same-day discharge lacks the assessment of hemoglobin and related indicators. Inpatients undergo more medical index monitoring and elaborate treatments. This study showed that outpatient TJA was associated with less surgical-related pain than inpatient TJA. We speculate that inpatients were given more detailed multimodal pain management than were same-day discharge outpatients.

## Conclusion

Outpatient TJA has advantages over inpatient TJA in THA total complications, THA readmissions, TJA readmissions, TJA stroke/cerebrovascular incidents, and TJA blood transfusion at 30 days and in THA readmissions, TJA total complications, and TJA surgical-related pain at 90 days. The remaining parameters presented comparable outcomes between outpatient and inpatient TJA. Overall, outpatient total knee and hip arthroplasty provide comparable and even better clinical outcomes than inpatient operations for well-selected patients. Multicenter randomized controlled trials with large samples are needed to provide stronger evidence in the future.

## Data Availability

The original contributions presented in the study are included in the article/Supplementary Material, further inquiries can be directed to the corresponding author/s.
