# Supplementary material for: Outpatient total knee and hip arthroplasty present comparable and even better clinical outcomes than inpatient operation
Source: Front Surg. 2022 Sep 6;9:833275. doi: 10.3389/fsurg.2022.833275 (PMC9485540; doi:10.3389/fsurg.2022.833275)
Supplement: Supplementary file 1 [file Table_2_v1.docx]

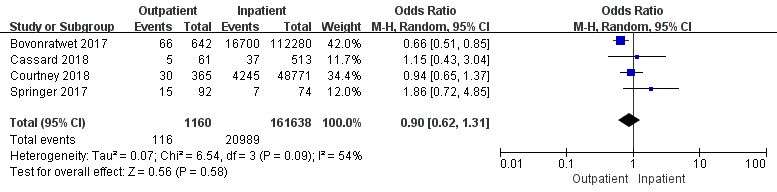


Figure. S1. Comparison of 30-day total complications between outpatient and inpatient TKA.


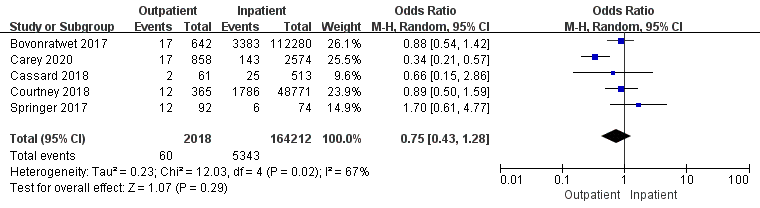


Figure. S2. Comparison of 30-day readmissions between outpatient and inpatient TKA.


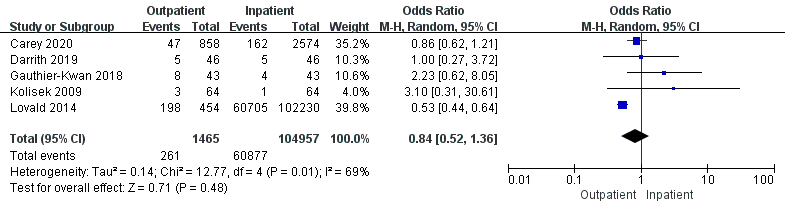


Figure. S3. Comparison of 90-day total complications between outpatient and inpatient TKA.


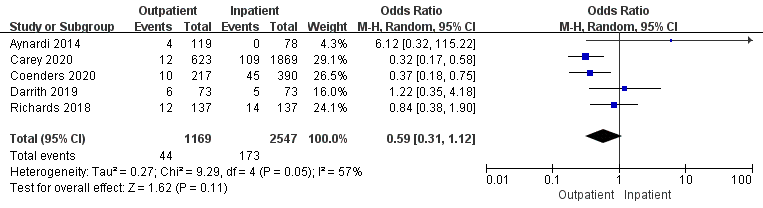


Figure. S4. Comparison of 90-day total complications between outpatient and inpatient THA.


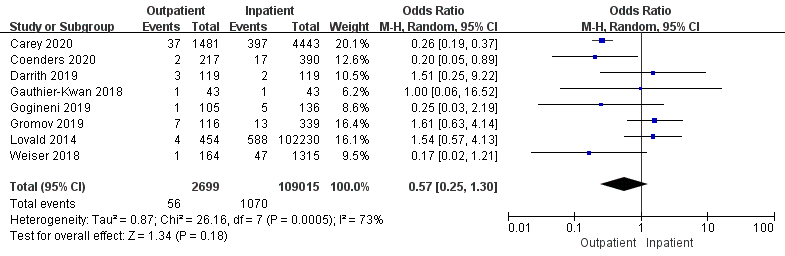


Figure. S5. Comparison of 90-day readmissions between outpatient and inpatient TJA.


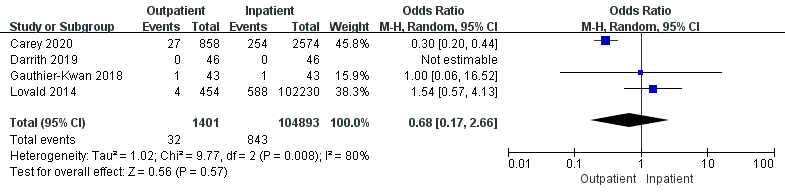


Figure. S6. Comparison of 90-day readmissions between outpatient and inpatient TKA.


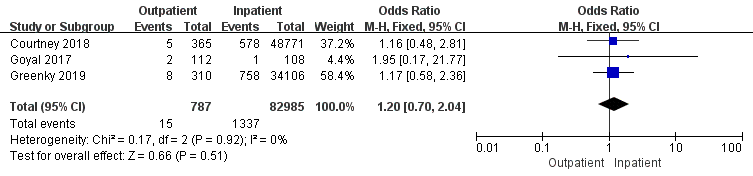


Figure. S7. Comparison of 30-day reoperations between outpatient and inpatient TJA.


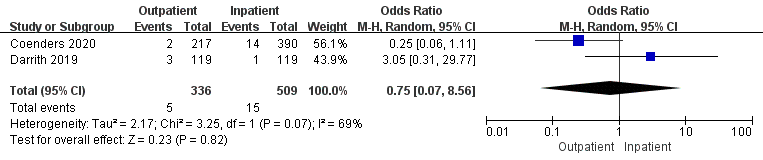


Figure. S8. Comparison of 90-day reoperations between outpatient and inpatient TJA.


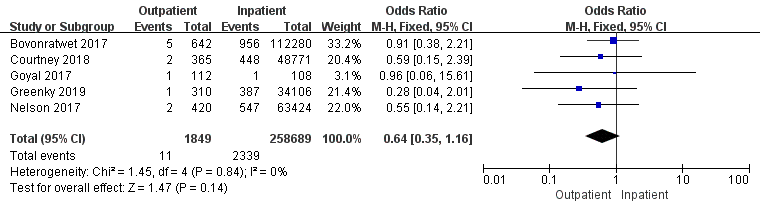


Figure. S9. Comparison of 30-day surgical site infections between outpatient and inpatient TJA.


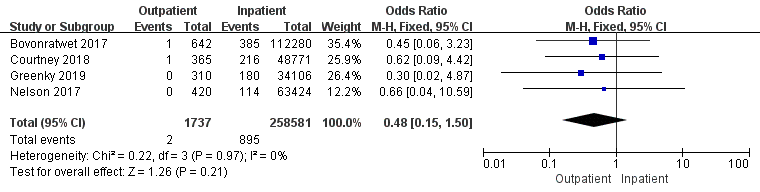


Figure. S10. Comparison of 30-day pneumonia between outpatient and inpatient TJA.


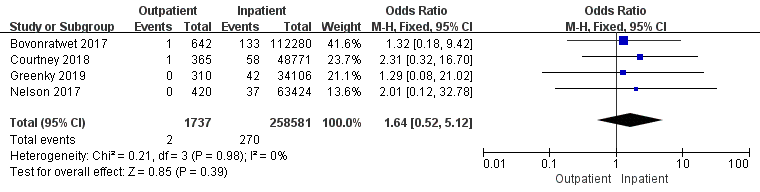


Figure. S11. Comparison of 30-day renal insufficiency cases between outpatient and inpatient TJA.


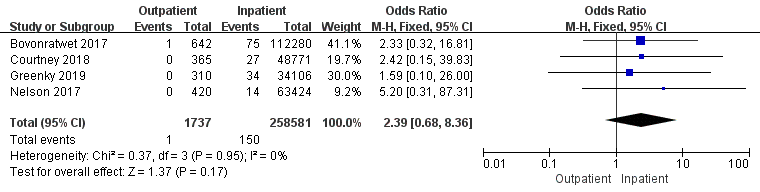


Figure. S12. Comparison of 30-day renal failure cases between outpatient and inpatient TJA.


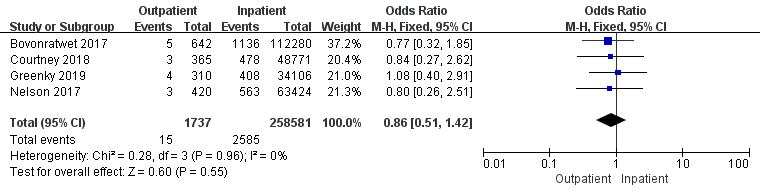


Figure. S13. Comparison of 30-day urinary tract infections between outpatient and inpatient TJA.


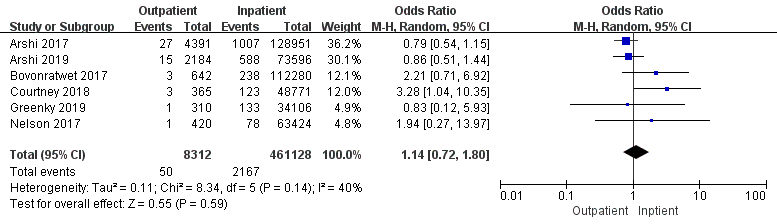


Figure. S14. Comparison of 30-day myocardial infarctions between outpatient and inpatient TJA.


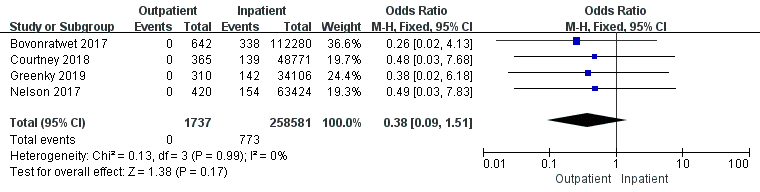


Figure. S15. Comparison of 30-day sepsis/septic shock cases between outpatient and inpatient TJA.


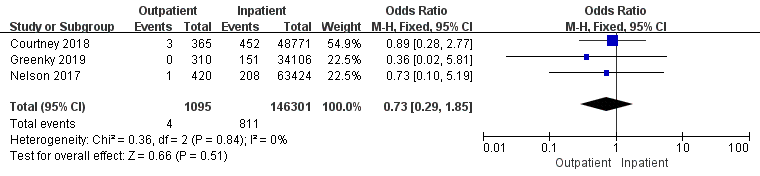


Figure. S16. Comparison of 30-day deep vein thrombosis cases between outpatient and inpatient TJA.


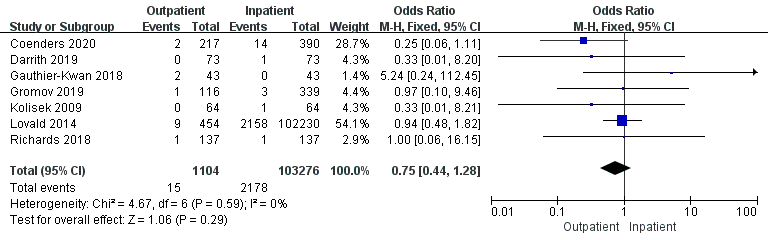


Figure. S17. Comparison of 90-day surgical site infections between outpatient and inpatient TJA.


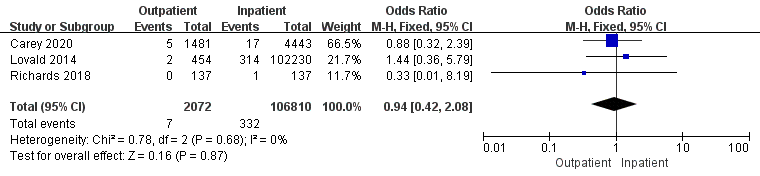


Figure. S18. Comparison of 90-day revisions between outpatient and inpatient TJA.


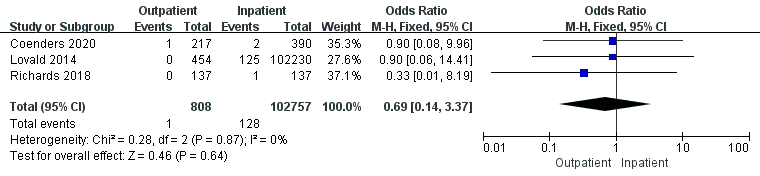


Figure. S19. Comparison of 90-day periprosthetic fractures between outpatient and inpatient TJA.


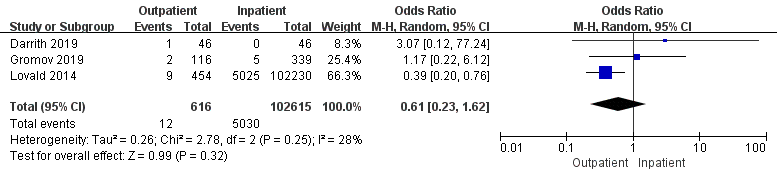


Figure. S20. Comparison of 90-day deep vein thrombosis cases between outpatient and inpatient TJA.


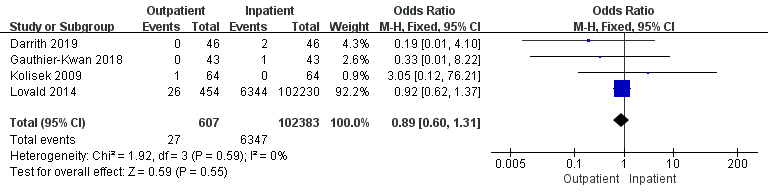


Figure. S21. Comparison of 90-day arthrofibrosis cases between outpatient and inpatient TJA.
